# Supplementary material for: The Effectiveness and Toxicity of Frameless CyberKnife Based Radiosurgery for Parkinson’s Disease—Phase II Study
Source: Biomedicines. 2023 Jan 20;11(2):288. doi: 10.3390/biomedicines11020288 (PMC9952894; doi:10.3390/biomedicines11020288)
Supplement: Supplementary file 1 [file biomedicines-11-00288-s001.zip › biomedicines-2130743-supplementary.pdf]

Table S1. Treatment results of particular patients based on neurologist examination (N) and patients self-evaluation (P).

Time of control visit	Total dose	FU duration (months)	3 months FU visit	6 months FU visit	12 months FU visit	Last follow-up visit	Time to tremor progression
Patient							
1	70 Gy	30	Improvement (N) Improvement (P)	***Progression (N) No improvement (P)***	No change (N) No improvement (P)	No change (N) No improvement (P)	6 months
2	70 Gy	36	Improvement (N) Improvement (P)	Improvement (N) Improvement (P)	No change (N) Improvement (P)	No change (N) Improvement (P)	Not occurred
3	70 Gy	43	Improvement (N) Improvement (P)	No change (N) Improvement (P)	No change (N) Improvement (P)	***Progression (N) No improvement (P)***	36 months
4**	75 Gy	38	No change (N) Improvement (P)	No data	No change (N) Improvement (P)	Improvement (N) Improvement (P)	Not occurred
5**	75 Gy	31	Improvement (N) Improvement (P)	No change (N) Improvement (P)	No change (N) Improvement (P)	Improvement (N) Improvement (P	Not occurred
6	75 Gy	25	Improvement (N) Improvement (P)	No change (N) Improvement (P)	No change (N) Improvement (P)	No change (N) Improvement (P)	Not occurred
7	*80 Gy*	*13*	*No change (N) No improvement (P)*	***Progression (N) No improvement (P)***	***Progression (N) No improvement (P)***	***Progression (N) No improvement (P)***	***Not responded to treatment***
8*	80 Gy	30	**Progression** (N) No improvement (P)	No change (N) No improvement (P)	Improvement (N) Improvement (P)	***Progression (N) No improvement (P)***	3 months and 30 months (after improvement period between 3 to 24 months after SRS)
9	80 Gy	6	Improvement (N) Improvement (P)	Improvement (N) Improvement (P)	No data	Improvement (N) Improvement (P)	Not occurred
10	85 Gy	25	Improvement (N)	No change (N) Improvement (P)	No change (N) Improvement (P)	No change (N) Improvement (P)	Not occurred

			Improvement (P)				
11**	85 Gy	35	No change (N)<br>Improvement (P)	No change (N)<br>Improvement (P)	No change (N)<br>Improvement (P)	No change (N)<br>Improvement (P)	Not occurred
12*	85 Gy	19	Improvement (N)<br>Improvement (P)	No change (N)<br>Improvement (P)	Improvement (N)<br>Improvement (P)	No change (N)<br>Improvement (P)	Not occurred
13	90 Gy	10	Improvement (N)<br>Improvement (P)	No change (N)<br>Improvement (P)	No data	No change (N)<br>Improvement (P)	Not occurred
14	90 Gy	18	Improvement (N)<br>Improvement (P)	***Progression (N)***<br>***No improvement (P)***	No change (N)<br>No improvement (P)	***Progression (N)***<br>***No improvement (P)***	5 months
15	95 Gy	23	Improvement (N)<br>Improvement (P)	No change (N)<br>Improvement (P)	***Progression (N)***<br>***No improvement (P)***	No change (N)<br>No improvement (P)	9 months
16	*95 Gy*	*14*	*No data*	*No data*	***Progression (N)***<br>***No improvement (P)***	***Progression (N)***<br>***No improvement (P)***	***Not responded to treatment***
17*	95 Gy	14	Improvement (N)<br>Improvement (P)	No data	Improvement (N)<br>Improvement (P)	Improvement (N)<br>Improvement (P)	Not occurred
18	100 Gy	9	No change (N)<br>No improvement (P)	No change (N)<br>No improvement (P)	No data	No change (N)<br>No improvement (P)	Not occurred
19*	100 Gy	12	No data	No data	No change (N)<br>Improvement (P)	No change (N)<br>Improvement (P)	Not occurred
20	100 Gy	19	No change (N)<br>Improvement (P)	Improvement (N)<br>Improvement (P)	No change (N)<br>Improvement (P)	No change (N)<br>No improvement (P)	Not occurred
21	105 Gy	4	No change (N)<br>Improvement (P)	No data	No data	No change (N)<br>Improvement (P)	Not occurred

* Late (8,12,17,19) and **very late (4,5,11) responders - maximum improvement observed at 12 to 14 and 24 to 35 months after the treatment, respectively
